# Novel resistance functions uncovered using functional metagenomic investigations of resistance reservoirs

**DOI:** 10.3389/fmicb.2013.00145

**Published:** 2013-06-07

**Authors:** Erica C. Pehrsson, Kevin J. Forsberg, Molly K. Gibson, Sara Ahmadi, Gautam Dantas

**Affiliations:** ^1^Center for Genome Sciences and Systems Biology, Washington University School of MedicineSt. Louis, MO, USA; ^2^Department of Pathology and Immunology, Washington University School of MedicineSt. Louis, MO, USA; ^3^Department of Biomedical Engineering, Washington UniversitySt. Louis, MO, USA

**Keywords:** functional metagenomics, antibiotic resistance, bifunctional resistance gene, environmental resistance, resistance reservoir, transferable resistance

## Abstract

Rates of infection with antibiotic-resistant bacteria have increased precipitously over the past several decades, with far-reaching healthcare and societal costs. Recent evidence has established a link between antibiotic resistance genes in human pathogens and those found in non-pathogenic, commensal, and environmental organisms, prompting deeper investigation of natural and human-associated reservoirs of antibiotic resistance. Functional metagenomic selections, in which shotgun-cloned DNA fragments are selected for their ability to confer survival to an indicator host, have been increasingly applied to the characterization of many antibiotic resistance reservoirs. These experiments have demonstrated that antibiotic resistance genes are highly diverse and widely distributed, many times bearing little to no similarity to known sequences. Through unbiased selections for survival to antibiotic exposure, functional metagenomics can improve annotations by reducing the discovery of false-positive resistance and by allowing for the identification of previously unrecognizable resistance genes. In this review, we summarize the novel resistance functions uncovered using functional metagenomic investigations of natural and human-impacted resistance reservoirs. Examples of novel antibiotic resistance genes include those highly divergent from known sequences, those for which sequence is entirely unable to predict resistance function, bifunctional resistance genes, and those with unconventional, atypical resistance mechanisms. Overcoming antibiotic resistance in the clinic will require a better understanding of existing resistance reservoirs and the dissemination networks that govern horizontal gene exchange, informing best practices to limit the spread of resistance-conferring genes to human pathogens.

## Overview

It is estimated that infection with antibiotic-resistant pathogens incurs over $25 billion in societal and healthcare costs annually in the United States (CDC, 2011). More than 70% of hospital-acquired bacterial infections are currently resistant to at least one of the major antibiotics used as standard treatment (Stone, 2009), and patient deaths as a result of hospital-acquired infections have increased by over 675% during the past 20 years (NIAID, 2012). As the incidence and spectrum of antibiotic resistance increases (Arias and Murray, 2009), antibiotic development has slowed to a trickle (Spellberg et al., 2008), further hindering the effectiveness of antibiotics to treat infectious disease. The majority of acquired antibiotic resistance genes in bacterial pathogens are obtained via horizontal gene transfer (HGT) (Ochman et al., 2000), likely with environmental origins (Benveniste and Davies, 1973; Wright, 2010; D'Costa et al., 2011). Accordingly, an increasing impetus has been placed on cataloging antibiotic resistance reservoirs (Allen et al., 2009b, 2010), determining how resistance genes are most readily transferred to the clinic (Marshall and Levy, 2011; Smillie et al., 2011), and identifying resistance mechanisms heretofore unseen in clinical settings (Jeon et al., 2011; Tao et al., 2012).

## Traditional mechanisms of antibiotic resistance

An antibiotic-resistant phenotype can be either an acquired trait or intrinsic to the bacterium in question. In the case of intrinsic resistance, antibiotic therapy is rendered ineffective due to a pre-existing physiological trait of the species, such as reduced accessibility to or absence of a drug target (Alekshun and Levy, 2007; Martinez, 2008; Davies and Davies, 2010; Dantas and Sommer, 2012). Examples of intrinsic antibiotic resistance include vancomycin tolerance in Gram-negative bacteria (the outer cell membrane reduces access to the peptidoglycan target) (Arthur and Courvalin, 1993) and biofilm formation in numerous organisms (perhaps providing resistance via reduced drug penetration and/or altered microenvironments) (Stewart and Costerton, 2001). Conversely, acquired antibiotic resistance stems from the expression of a specific resistance gene and is commonly the result of *de novo* mutation or the acquisition of resistance-conferring genes on mobile genetic elements (e.g., plasmids, transposons, integrons) (Walsh, 2003). The antibiotic resistance genes present in a microbial community that are capable of transfer to a new host are collectively referred to as the “transferable resistome.” Intrinsic resistance is, by definition, limited to the context of the parent organism, whereas acquired resistance represents a more flexible phenotype, and its prevalence is more immediately responsive to selection pressure (Martinez, 2008). As nearly all infectious bacteria were antibiotic-susceptible prior to the introduction of antibiotic therapy (Houndt and Ochman, 2000; Davies and Davies, 2010), the exceeding majority of resistance in human pathogens is acquired, either through mutation or HGT (Alekshun and Levy, 2007). This resistance represents a diversity of biochemical mechanisms that break down into three general categories (Walsh, 2000, 2003): (1) inactivation of the antibiotic, (2) reducing intracellular antibiotic concentration through efflux or permeability barriers, and (3) altering the cellular target of the antibiotic, reducing their association.

Perhaps the most intuitive of resistance mechanisms, antibiotic inactivation, is sub-categorized into two groups: enzymes that inactivate drugs via degradation (e.g., the β-lactamases) vs. those that function via chemical modification. The β-lactamases are characterized by their ability to cleave the four-membered ring present in all β-lactam antibiotics and are some of the best-studied and widely-distributed antibiotic resistance genes (for review, see Jacoby and Munoz-Price, 2005). These enzymes confer high-level antibiotic resistance and are found associated with mobile DNA elements and integrated into bacterial chromosomes. β-lactamases function via either a serine active site or metal cation cofactor (Jacoby and Munoz-Price, 2005) and can be found across bacterial phyla. Antibiotic-modifying enzymes are also phylogenetically widespread, as well as mechanistically diverse. These enzymes can confer tolerance toward numerous drugs, including the aminoglycoside (Davies and Wright, 1997), tetracycline (Yang et al., 2004), amphenicol (Schwarz et al., 2004), and macrolide-lincosamide-streptogramin (Weisblum, 1998) antibiotics, typically functioning via covalent modification of the drug with some functional moiety (e.g., acetyl, phosphoryl, nucleotidyl, glycosyl, and hydroxyl groups) (Alekshun and Levy, 2007).

The intracellular concentration of any given antibiotic can be reduced by either efflux mechanisms to remove the drug from the cytosol or permeability barriers that limit the drug's uptake. Many antibiotics have poor activity against Gram-negative pathogens due to efflux systems (Levy, 1992), most notably the RND superfamily transporters (Li and Nikaido, 2004, 2009). Other major families of efflux systems include the MFS, SMR, and ABC superfamily transporters, which are present in both Gram-negative and -positive organisms (Li and Nikaido, 2004, 2009). Although commonly chromosomal, many efflux systems are found on plasmids and other mobile elements and can confer drug-specific, class-specific, or multidrug resistance (Poole, 2005). Some permeability barriers, such as the Gram-negative outer membrane (Arthur and Courvalin, 1993), represent intrinsic antibiotic resistance, while in other instances, permeability barriers are acquired. Examples include multidrug-resistance via the altered expression of Gram-negative porin proteins (e.g., OmpF in *Escherichia coli* and OprD in *Pseudomonas*) (Delcour, 2009) and glycopeptide resistance due to thickened Gram-positive cell walls (Cui et al., 2006).

Antibiotic resistance via cellular target modification often occurs via chromosomal mutation and represents a common means by which the fluoroquinolone, sulfonamide, and trimethroprim antibiotics, among others, are tolerated (Alekshun and Levy, 2007). In the case of the fluoroquinolones, mutations to a variety of residues within the quinolone-resistance-determining-region (QRDR) of the DNA gyrase GyrA or topoisomerase IV ParC/GrlA prevent the interaction of the synthetic antibiotic with its target, facilitating resistance (Hooper, 1999). Importantly, resistance to fluoroquinolones via mutation is typically recessive, suppressing the acquisition of a resistant phenotype in the presence of wild-type GyrA or ParC/GrlA and thus preventing widespread horizontal dissemination of resistant gene variants (Wolfson and Hooper, 1989; Soussy et al., 1993). However, a plasmid-borne GyrA protection protein, Qnr, has been discovered that confers low-level fluoroquinolone resistance (Tran and Jacoby, 2002) and can potentiate the incidence of QRDR mutations, which combined provide high levels of resistance (Jacoby, 2005). Both the sulfonamides and trimethroprim competitively inhibit enzymes within the folate biosynthesis pathway: mutations to the dihydropteroate synthase and dihydrofolate reductase (DHFR) enzymes can reduce affinity for sulfonamides and trimethroprim, respectively, and provide tolerance to the antibiotics (Huovinen et al., 1995). In addition to arising *de novo*, both sulfonamide- and trimethroprim-resistant enzymes are present on mobile DNA elements, providing resistance to numerous bacteria via HGT (Alekshun and Levy, 2007). Other highly mobile mechanisms of target-modification include vancomycin resistance via the modification of peptidoglycan precursors (Bugg et al., 1991), aminoglycoside resistance via methylation of the 16S rRNA subunit (Galimand et al., 2003), and macrolide resistance from 23S rRNA methylases (Zhanel et al., 2001).

## Reservoirs of transferable antibiotic resistance

Research on antibiotic resistance over the past 70 years has focused on traditionally pathogenic bacteria isolated in a clinical setting and the role of antibiotic resistance genes already present in those species (Sommer et al., 2009; Davies and Davies, 2010). The resistance phenotype, however, is an ancient function of environmental bacteria (D'Costa et al., 2011), despite being largely absent from human pathogens prior to the antibiotic age (Hughes and Datta, 1983; Houndt and Ochman, 2000), with estimates that β-lactamases have existed for over 2 billion years (Hall and Barlow, 2004; Hall et al., 2004). Moreover, diverse mechanisms of antibiotic resistance have been discovered in nearly all environments (D'Costa et al., 2006; Allen et al., 2010; Davies and Davies, 2010; Wright, 2010); seemingly each new metagenomic study uncovers numerous examples of resistance genes previously unreported in public databases (Allen et al., 2009b; Sommer et al., 2009; Donato et al., 2010; Forsberg et al., 2012). In short, the diversity and abundance of antibiotic resistance in commensal microbiota and environmental settings dwarfs that which is seen in the context of human pathogens.

Importantly, environmental antibiotic resistance is not only widespread, but also represents the likely origins of the resistance seen in human pathogens. It has been known for 40 years that environmental bacteria share the same resistance mechanisms as those seen in pathogens (Benveniste and Davies, 1973), with documented examples of environmental resistance genes moving from natural settings into human pathogens (Poirel et al., 2002, 2005). It is becoming increasingly evident that human pathogens and environmental organisms share antibiotic resistance genes; a recent study described seven resistance genes from non-pathogenic soil organisms, conferring tolerance to five antibiotic classes, with perfect identity to genes from phylogenetically and geographically diverse pathogens (Forsberg et al., 2012). Given the staggering diversity of environmental resistance, high adaptability of bacteria, and strong selection pressure for antibiotic resistance, the question of antibiotic resistance is “not a matter of if but only a matter of when” (Walsh, 2000).

To understand *when* novel resistance will appear in human pathogens and perhaps diminish its impact, one must understand *how* new resistance genes are most frequently acquired by pathogenic bacteria. Since the answer is, most commonly, *via HGT* (Hughes and Datta, 1983; Ochman et al., 2000; Alekshun and Levy, 2007), understanding the complement of resistance genes most likely to be transferred to pathogens is crucial to predicting resistance acquisition. Although cataloging the repertoire of resistance genes on Earth remains a prohibitively large undertaking, techniques for interrogating the resistance properties of complex microbial communities exist and are being applied toward the identification of diverse and novel resistance from numerous settings. Importantly, these studies are focused not only on environmental locales, but also on the resistomes associated with human and animal microbiota (Shoemaker et al., 2001; Sommer et al., 2010b; Sommer and Dantas, 2011). Although antibiotic resistance may have its origins in the environment (Benveniste and Davies, 1973; Wright, 2010; D'Costa et al., 2011), the commensal resistome shares many resistance genes with both pathogens (Sommer et al., 2010b) and environmental organisms (Forsberg et al., 2012) and represents a likely route through which these populations exchange resistance genes (Smillie et al., 2011; Sommer and Dantas, 2011). Focused efforts to understand how pathogens acquire antibiotic resistance will require a greater appreciation for the diversity of resistance genes and in which environments and under what conditions these resistance genes are most accessible to human pathogens.

## Interrogating antibiotic resistance properties in complex microbial communities

Traditionally, either culture-based (D'Costa et al., 2006) or PCR-based approaches (Perez-Perez and Hanson, 2002) have been used to study antibiotic resistance properties from microbial communities. While both techniques have led to major discoveries (Galan et al., 2013), both have inherent limitations that have contributed to an under-sampling of resistance genes from diverse microbial habitats. The majority of bacteria remain recalcitrant to culturing (Daniel, 2005) and are therefore not interrogated when culture-dependent techniques are employed. Additionally, linking a resistance phenotype to a causal genotype is a time-consuming process, often necessitating experimental scope to be limited to a small number of organisms, rather than whole communities. PCR screens are an effective means to identify or quantify resistance genes of known sequence, circumventing the need for culture (Knapp et al., 2010), but are only able to detect previously described genes and often require expression cloning and subsequent experimentation to verify function. In addition, annotation of antibiotic resistance genes in shotgun-sequenced microbial communities has proven challenging, as homology-based functional gene comparisons often fail due to low sequence similarity to previously discovered resistance genes, and *in silico* analyses are unable to confirm resistance function.

**Figure 1:**
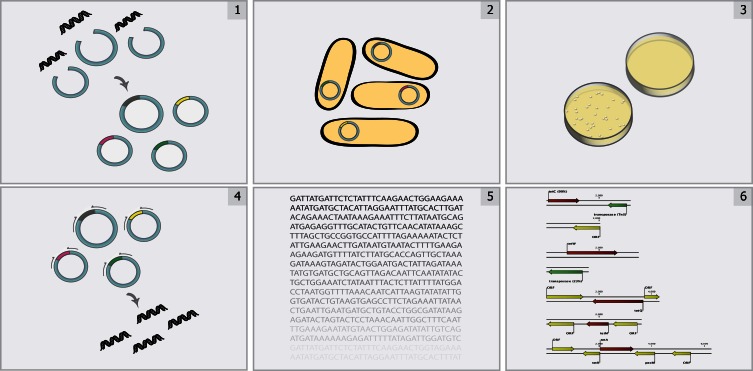
**Overview of functional metagenomic selections.** Total metagenomic DNA is extracted from a microbial community sample, sheared, and ligated into an expression vector (Step 1) and is subsequently transformed into a suitable library host (Step 2) to create a metagenomic library. The library is then plated on media containing antibiotics inhibitory to the wild-type host (Step 3) to select for metagenomic fragments conferring antibiotic resistance. Metagenomic fragments present in colonies growing on antibiotic selection media are then PCR-amplified (Step 4) and sequenced using either traditional Sanger sequencing or next-generation sequencing methods (Step 5). Finally, reads are assembled and annotated in order to identify the causative antibiotic resistance genes (Step 6).

In contrast with standard techniques, *functional metagenomics* is a culture- and sequence-independent means of identifying transferrable antibiotic resistance in complex metagenomes. This method (Figure 1) involves shotgun-cloning total community DNA into an expression vector and transforming the library into an indicator host (commonly the model organism *E. coli*). The resulting transformants are then selected for the desired function (e.g., antibiotic resistance), and metagenomic DNA fragments are sequenced and annotated to identify causal survival-conferring genes (Allen et al., 2009b; Sommer et al., 2009). Functional metagenomics offers three classical advantages for the unbiased interrogation of complex resistomes (Daniel, 2005; Sommer and Dantas, 2011): (1) no need to culture organisms, (2) no required knowledge of resistance gene sequence, and (3) direct association between a genotype and a demonstrated resistance phenotype. Additionally, functional metagenomic selections specifically identify those genes within a metagenome capable of conferring antibiotic tolerance to the indicator host when expressed exogenously (i.e., they distinguish transferrable resistance from intrinsic resistance) (Dantas and Sommer, 2012). Recent improvements to the throughput of functional metagenomics (Forsberg et al., 2012) unlock the potential for the experiments of scale needed identify the specific sequences, and environments, most readily able to confer resistance to human pathogens, frequently represented by the opportunistic pathogen *E. coli*.

## Novel resistance mechanisms uncovered by functional metagenomic selections

The following sections review novel resistance mechanisms and genes uncovered by functional metagenomic selections; these studies are summarized in Table 1.

**Table 1:** **Functional metagenomic investigations of antibiotic resistance reservoirs**.

**Resistance reservoir**	**Date**	**Novel resistance function identified**	Total DNA queried^*^	**References**
**FUNCTIONAL METAGENOMIC INVESTIGATIONS OF ENVIRONMENTAL RESERVOIRS**				
Soil (remnant oak savannah)	2004	First use of functional metagenomic selections to investigate environmental antibiotic resistance; Nine novel aminoglycoside-resistance genes	5.4 GB	Riesenfeld et al., 2004
Activated sludge	2008	Two novel bleomycin-resistance genes	3.2 GB	Mori et al., 2008
Remote Alaskan soil	2009	13 novel β-lactamases; First discovery of bifunctional β-lactamase	12.4 GB	Allen et al., 2009b
Gypsy moth larvae midgut isolates	2009	*ramA* (AraC transcriptional regulator)	0.3 GB	Allen et al., 2009a
Remote Alaskan soil	2010	*pexA* (novel chloramphenicol transporter)	13.2 GB	Lang et al., 2010
Activated sludge	2010	Six novel chloramphenicol-modifying resistance genes; Novel aminoglycoside-resistance gene	1.9 GB	Parsley et al., 2010
Soil (apple orchard)	2010	Nine novel β-lactamases; Three novel aminoglycoside-resistance genes; A novel tetracycline efflux pump	13.4 GB	Donato et al., 2010
Alluvial soil	2011	Est136, chloramphenicol acetate esterase	1.6 GB	Tao, 2011; Tao et al., 2012
Soil (agricultural, nature reserve)	2011	*Tm8-3* (novel dihydrofolate reductase)	3.6 GB	Torres-Cortes et al., 2011
Wetland soil	2011	EstU1, family VIII carboxylesterase	0.3 GB	Kim et al., 2008; Jeon et al., 2011
Gull gut microbiome	2011	31 β-lactam resistance genes of undetermined mechanism	20.5 GB	Martiny et al., 2011
Urban soil	2012	Novel MFS and ABC transporters; Five novel aminoglycoside-resistance genes; ADP-ribosyltransferase longer than any previously discovered	2.8 GB	McGarvey et al., 2012
Multidrug-resistant soil isolates	2012	Novel β-lactamases; D-cycloserine efflux pump	2.6 GB	Forsberg et al., 2012
**FUNCTIONAL METAGENOMIC INVESTIGATIONS OF HUMAN-ASSOCIATED RESERVOIRS**				
Human oral microbiome	2003	Tetracycline-inactivating protein *tet(37)*	0.4–1.4 MB	Diaz-Torres et al., 2003
Human oral microbiome	2006	Tetracycline-resistance genes of undetermined mechanism	27.8 MB	Diaz-Torres et al., 2006
Human gut and oral microbiome	2009	10 new β-lactamase classes; Seven novel aminoglycoside-resistance genes	9.3 GB	Sommer et al., 2009
Pig gut microbiome	2009	Tetracycline-resistance genes *galE1* and *galE2* of undetermined mechanism	0.1 GB	Kazimierczak et al., 2009
Human gut microbiome	2012	Confirmed bifunctional aminoglycoside-resistance gene; Novel β-lactamase; Novel aminoglycoside-resistance gene	12.5 GB	Cheng et al., 2012

* For comparison, the genome size of E. coli K-12 substrain MG1655 is 4.64 MB.

### Genes highly divergent from known resistance genes

PCR-based screens for known resistance genes are able to detect novel variants with minor sequence differences, but due to the requirement for conserved primer binding sites, they are often unable to detect genes that have diverged significantly from the canonical example. In comparison, functional metagenomic selections for antibiotic resistance frequently identify genes that are less than 65% identical at the amino acid level to known resistance genes. In this way, functional metagenomics expands our knowledge of what sequence variants are tolerated in a resistance gene while preserving the resistance mechanism, as well as what mutations lead to expanded specificity profiles.

β-lactamases are one of the largest and best studied classes of resistance determinants, and yet, novel β-lactamases are regularly uncovered with sequence-independent techniques (Allen et al., 2009b; Sommer et al., 2009; Donato et al., 2010; Cheng et al., 2012; Forsberg et al., 2012). In the first functional metagenomic selection for antibiotic resistance in the human gut microbiome, Sommer et al. identified ten novel β-lactamase families whose eleven members were only 35–61% identical to known genes (Sommer et al., 2009). Based on phylogenetic analysis with PhyloPythia, (McHardy et al., 2007) these genes originated primarily from the Firmicutes and Bacteroidetes phyla, which comprise the majority of the human gut microbiota but have been undersampled because they are less readily culturable in aerobic environments than Proteobacteria. Functional selection for β-lactam resistance in metagenomic libraries constructed from remote Alaskan soil revealed 13 novel β-lactamases of all four Ambler classes that were ≤67% identical to any known, functionally characterized gene (Allen et al., 2009b). In the case of the class B β-lactamases, several genes appear to be more closely related to the ancestral gene of that class than to clinically isolated genes, providing context for the evolution of clinical isolates. The study also confirmed that overall sequence similarity does not necessarily confer similar drug susceptibility profiles, supporting discovery techniques that confirm the function of identified genes.

Functional metagenomic selections also enable the detection of highly diverse classes of resistance genes, defined by substrate specificity more than shared sequence identity. For instance, aminoglycoside 6′-N-acetyltransferases [AAC(6′)s] are difficult to identify via PCR because of their high sequence diversity (Riesenfeld et al., 2004). Sommer et al. identified six AAC(6′)s with less than 48% amino acid identity to known genes and a methyltransferase conferring resistance to sisomycin with only 26.3% shared identity. Riesenfeld et al. and other studies have identified many aminoglycoside resistance genes less than 70% identical to previously reported sequences (Riesenfeld et al., 2004; Donato et al., 2010; Torres-Cortes et al., 2011; Cheng et al., 2012; McGarvey et al., 2012). Similarly, two novel bleomycin resistance genes were isolated from an activated sludge metagenomic library (Mori et al., 2008). Bleomycin resistance proteins act primarily by sequestering the antibiotic through electrostatic interactions, so their sequences tolerate an extreme amount of divergence while still maintaining the resistance function. Nevertheless, the study confirmed that all known bleomycin resistance proteins contain a proline near the N-terminus and have an acidic pI of <5, improving computational annotation of this class. Finally, McGarvey et al. identified an ADP-ribosyltransferase that was much longer any previously characterized using functional metagenomic selections (McGarvey et al., 2012). This full-length gene may not have been identified by PCR-based screens, but its discovery, enabled by functional metagenomics, expands our knowledge of the sequence diversity tolerated within this resistance class.

### Resistance genes that would not have been predicted by sequence

A major limitation of sequence-based metagenomics is its restriction to antibiotic resistance genes that are recognizable as members of a previously characterized class. BLAST, the most commonly used annotation tool, requires a threshold of shared sequence identity for a gene to be considered a member of an established gene class. Therefore, by definition, this method limits the ability to identify novel resistance genes. In addition, while profile hidden Markov model (HMM)-based annotation provides a sensitive, statistically sound analysis method capable of identifying remote homologs, these models still rely on underlying multiple sequences alignments of previously characterized proteins. In addition, current profile HMM databases provide high-level classification of antibiotic resistance genes (e.g., “Beta-Lactamase,” rather than “TEM Beta-Lactamase”), providing little functional information about the gene and its potential resistance profile. Therefore, while these techniques have the potential to identify genes that have low sequence identity to known resistance genes, in the absence of additional functional confirmation, they cannot identify novel resistance mechanisms or verify that sequence variants are functional.

Functional metagenomics identifies resistance-conferring elements without prior knowledge of the sequence, circumventing this limitation. For instance, in a selection for tetracycline resistance encoded by the human oral metagenome, Diaz-Torres et al. discovered a novel tetracycline resistance gene, *tet(37)* (Diaz-Torres et al., 2003). *In vitro* analysis indicated that *tet(37)* inactivates tetracycline, making it one of only three tetracycline resistance proteins to utilize this mechanism (Thaker et al., 2010). *tet(37)* resembles flavoproteins, oxidoreductases, and NAD(P)-requiring enzymes in sequence and conserved motifs, but has no identity to *tet(X)*, the first tetracycline-inactivating gene identified. It is therefore unlikely that computational annotation would have identified it as a resistance gene (Diaz-Torres et al., 2003).

Similarly, a study of soil microbiota identified a dihydrofolate-reducing gene, *Tm8-3*, that resembles 3-oxoacyl-(acyl-carrier-protein) reductases but not *dhfr*, the target of trimethoprim (Torres-Cortes et al., 2011). A screen of an activated sludge metagenome also identified six resistance genes that appear to inactivate chloramphenicol through enzymatic modification, but which share no significant identity with known chloramphenicol acetyltransferases (Parsley et al., 2010). Although these genes may become important for clinical resistance in the future, their existence would likely have been overlooked without high-throughput functional selections independent of previous sequence knowledge.

Drug efflux is a widespread resistance mechanism common to many antibiotic classes, but individual transporters identified through computational annotation methods cannot be assigned antibiotic efflux properties without functional validation. Small changes in protein structure can change the drug specificity profile of an efflux pump and confer or eliminate resistance. Therefore, functional metagenomic selections are an attractive alternative for high-throughput characterization of transporters with tentative resistance annotations (Torres-Cortes et al., 2011). For instance, in their study of soil isolate metagenomes, Forsberg et al. identified a novel gene with only low identity to a drug/metabolite transporter. Were it not for functional selection of D-cycloserine resistance, this putative transporter would never have been identified as an antibiotic resistance gene (Forsberg et al., 2012). McGarvey et al. identified five novel MFS transporters and two ABC transporters from an urban soil that conferred resistance to tetracycline, chloramphenicol, or trimethoprim (McGarvey et al., 2012). Lang et al. successfully identified *pexA*, a novel amphenicol MFS transporter, despite its low identity (33%) to other drug resistance transporters, including any known chloramphenicol exporters (Lang et al., 2010).

### Bifunctional enzymes

Broad-spectrum resistance as a result of bifunctional resistance genes, the fusion of two complementary enzymatic functions into a single gene, is recognized as an increasing occurrence in many pathogens (Kim et al., 2006; Perez et al., 2007; Chandrakanth et al., 2008). Functional metagenomics provides an opportunity to identify fusion genes that are functionally active against multiple classes of antibiotic agents from natural resistance reservoirs and commensal organisms, and which have the potential to appear in clinically relevant bacteria.

Recently, functional metagenomic selections of a remote Alaskan soil revealed a novel bifunctional β-lactamase (Allen et al., 2009b). The 609-amino-acid protein is nearly double the length of a typical β-lactamase and is a natural fusion of genes for two different β-lactamase subclasses. The C-terminal domain (356 residues) aligns with class C β-lactamases, while the N-terminal domain (253 residues) aligns with class D β-lactamases, each contributing to the resistance profile of the full-length gene. The class C homologue confers resistance to cephalexin, while the class D homologue is responsible for resistance to amoxicillin, ampicillin, and carbenicillin. The bifunctional fusion gene therefore expands the resistance profile of the full-length gene beyond what either domain is responsible for alone (Allen et al., 2009b).

While this was the first bifunctional β-lactamase to be discovered, bifunctional resistance has been documented in the past, particularly against the aminoglycoside antibiotics (Daigle et al., 1999; Centrón and Roy, 2002; Dubois et al., 2002; Mendes et al., 2004; Robicsek et al., 2006). The most common mechanism of aminoglycoside resistance is deactivation of the molecule through modification by cytoplasmic enzymes: aminoglycoside acetyltransferases (AACs), aminoglycoside phosphotransferases (APHs), or aminoglycoside nucleotidyltransferases (ANTs). Four bifunctional enzymes, combining two complementary aminoglycoside-modifying enzymes as separate domains into a single open reading frame, significantly broadening the resistance profile, are known. Ferretti et al. reported the first bifunctional enzyme, AAC(6′)/APH(2″), demonstrating that it is capable of both acetylation (ACC activity found in N-terminal domain) and phosphorylation (APH activity found in C-terminal domain) of aminoglycosides (Ferretti et al., 1986). This bifunctional enzyme was also discovered in a functional metagenomics study of the human gut microbiota (Cheng et al., 2012). The ability to doubly modify aminoglycosides enables this enzyme to confer resistance to nearly all clinically relevant aminoglycosides except streptomycin and spectinomycin (Daigle et al., 1999). Similarly, the bifunctional resistance enzymes characterized as an AAC(3)-Ib/AAC(6′)-Ib′ (Dubois et al., 2002), an ANT(3″)-Ii/AAC(6′)-IId (Centrón and Roy, 2002), and an AAC(6′)-30/AAC(6′)-Ib′ (Mendes et al., 2004) all exhibit an expanded resistance profile by combining two different aminoglycoside-modifying enzymes into a single gene.

AAC(6′)-Ib-cr is the only bifunctional resistance enzyme identified capable of conferring resistance to two different structural classes of antibiotics, aminoglycosides and fluoroquinolones (Vetting et al., 2008). A variant of a common aminoglycoside acetyltransferase AAC(6′)-Ib, AAC(6′)-Ib-cr is also capable of N-acetylation of fluoroquinolones. Only two codon changes in the original enzyme are responsible for the fluoroquinolone-resistance phenotype (Vetting et al., 2008).

The merger of two genes with complementary enzymatic activities represents a novel mechanism for overcoming the increasing challenge by antibiotics, resulting in extremely broad resistance to entire antibiotic structural classes. It is likely that more examples of bifunctional resistance exist in the environment, and functional metagenomics provides a powerful technique for the discovery of functionally active bifunctional enzymes that may have great clinical implications.

### Unusual mechanisms of resistance

Functional metagenomic selections have the potential to identify another important class of resistance genes, those that have a primary role in the cell other than antibiotic resistance. This includes housekeeping genes whose overexpression provides resistance, general stress response transcription factors, and enzymes with promiscuous activity.

Selections for trimethoprim resistance, for example, frequently identify genes encoding DHFR, the cellular target of trimethoprim (Torres-Cortes et al., 2011; McGarvey et al., 2012). Cloning a *dhfr* homolog into a high copy-number plasmid results in overexpression of DHFR, which sequesters the antibiotic (Flensburg and Skold, 1987). McGarvey et al. identified 19 highly divergent DHFRs from a soil metagenome. The proteins shared only 7–44% amino acid identity in pairwise alignments, but a subset selected for further investigation conferred similar levels of trimethoprim resistance on the host when cloned into the same position of a high-copy number vector (McGarvey et al., 2012). Although clinical isolates of *E. coli* that massively overproduce DHFR as a result of promoter mutations and intrinsically resistant *dhfr* homologs have been identified (Flensburg and Skold, 1987), in most instances, resistance conferred by overexpression of a heterologous gene is likely independent of its ability to replace the host homolog. Rather, the introduced gene may sequester enough of the antibiotic to allow the native homolog to continue functioning. Selections for D-cycloserine resistance that identify D-alanine-D-alanine ligase, the target of the antibiotic, likely fall under the same class (Cheng et al., 2012). Creating and screening libraries in low-copy number vectors is essential in these instances to identify genes that are naturally resistant to the antibiotic, rather than simply selecting for overexpression.

In contrast, antibiotic resistance conferred by transcriptional regulators represents the successful interaction of a heterologous gene with existing host cellular pathways. The AraC transcriptional stress response regulators *marA*, *soxS*, and *rob* share a highly overlapping regulon and mediate low-level antibiotic resistance in *E. coli* and its close relative *E. fergusonii* (Alekshun and Levy, 1997; Martin and Rosner, 2002). As might be expected, these genes are often identified in functional metagenomic selections against chloramphenicol, tetracyclines, and other antibiotics when *E. coli* is the indicator host. A homolog of the AraC transcriptional regulator *ramA*, from Enterobacter sp., was also identified from a gypsy moth larvae gut isolate metagenomic library as conferring resistance to *E. coli* against multiple antibiotics of different classes when overexpressed (Allen et al., 2009a). These results provide valuable information about the ability of horizontally acquired genes from closely related phyla to incorporate into a cell at the regulatory level, a mechanism of antibiotic resistance that has been previously under-considered.

Certain genes identified by functional metagenomic selections are surprising in that they more closely resemble protein families that perform unrelated functions in the cell than previously identified resistance genes. They may represent instances of expanded substrate specificity, where mutation to a gene with an unrelated function confers resistance. By first screening a soil metagenomic library for esterase activity, Jeon et al. identified a family VIII carboxylesterase that hydrolyzes both esters and the amide bond of β-lactams, apparently utilizing the same catalytic site residues for both reactions (Jeon et al., 2011). The protein changes that enable it to also hydrolyze β-lactams are currently unidentified. A novel chloramphenicol acetate esterase, EstDL136, isolated from a soil metagenome, hydrolyzes the amide linkage of both chloramphenicol and its synthetic derivative florfenicol (Tao, 2011; Tao et al., 2012). Many of the surrounding genes in the metagenomic fragment are most closely related to Sphingomonadaceae, which are frequently considered for bioremediation for their ability to degrade many compounds (Stolz, 2009). EstDL136 may have originally evolved to detoxify another compound, later expanding its substrate specificity to chloramphenicol.

## Future directions and challenges

Functional metagenomic selections have proven to be an excellent technique for the discovery of novel antibiotic resistance mechanisms and genes encoded by varied environmental and human-associated microbial communities. As library sizes continue to increase, diverse indicator hosts are established, and techniques for improved functional selection and mechanistic determination are developed, functional metagenomics will only become better powered to explore the transferable resistome of complex microbial communities.

All of the metagenomic libraries discussed above were constructed in *E. coli*. The commercial availability of competent *E. coli* with extremely high transformation efficiencies has made this organism the preferred host for library construction, and these studies show that *E. coli* permits the heterologous expression of genes from many phyla. However, there are many clinically important classes of antibiotics that are intrinsically inactive against this Gram-negative species (e.g., glycopeptides, macrolides, oxazolidinones), prohibiting functional metagenomic selections in this host from identifying antibiotic resistance genes active against these classes. Development of a Gram-positive indicator host will represent a major step forward for the field (Riesenfeld et al., 2004; Sommer et al., 2009). This host must also exhibit a high transformation efficiency to enable the creation of metagenomic libraries large enough to permit the discovery of novel resistance genes and should lack restriction-modification systems or other host defenses that would prevent the expression of foreign DNA. *E. coli* mutants that mimic Gram-positive species through the alteration of cell membrane and wall structure are another possible alternative (Tamae et al., 2008).

The taxa from which antibiotic resistance genes can be expressed in *E. coli* are also limited by the ability of the host to recognize gene promoters and ribosome binding sites and translate them into functional proteins. Engineering *E. coli* to produce tRNA that recognize rare codons and alternative sigma factors will expand the range of taxa interrogated (Riesenfeld et al., 2004; Sommer et al., 2009, 2010a).

Finally, genes of unknown function should be further investigated to determine their mechanisms of resistance. Functional metagenomic selections that uncover a multitude of confirmed resistance genes in a high-throughput manner often require complementary biochemical experimentation to understand novel mechanisms of antibiotic resistance. Many metagenomic functional selections have identified resistance-conferring inserts whose mechanisms remain obscure because they cannot be determined based on sequence alone (Riesenfeld et al., 2004; Diaz-Torres et al., 2006; Kazimierczak et al., 2009; Martiny et al., 2011; McGarvey et al., 2012). These genes are excellent candidates for future functional characterization.

## Alternative strategies to combat antibiotic resistance

The depth and diversity of antibiotic resistance genes uncovered by functional metagenomic selections, many of which are associated with mobile genetic elements, brings to light the need for novel strategies to combat antibiotic-resistant pathogens. Alternative approaches that modulate the host immune response (e.g., immunomodulatory peptides, vaccination, therapeutic antibodies) or target the pathogen (e.g., anti-virulence initiatives, phage therapy, antibiotic potentiators) have been explored with some success (Planson et al., 2011; Pieren and Tigges, 2012).

For instance, recent studies have suggested engineered bacteriophage as an adjuvant to enhance the bactericidal activity of antibiotics and avoid evolution of resistance to either (Lu and Collins, 2009; Parracho et al., 2012). In this case, bacteriophage is engineered to repress non-essential genes (e.g., the SOS system) not directly targeted by antibiotics, weakening the cell and potentiating antibiotic activity. Other adjuvant compounds that inhibit intrinsic repair pathways or cell tolerance mechanisms are being explored (Fischbach, 2011).

Several permeabilizing products capable of disturbing the cell membrane have been identified, thus allowing antibiotics to penetrate the cells more efficiently. One such product is the low molecular-weight oligosaccharide nanomedicine OligoG, which is able to disturb multidrug-resistant bacteria (Khan et al., 2012).

Drug efflux pumps, both specific and multidrug-resistant, may be inhibited through a variety of strategies. These include interfering with the expression of a functional transporter at different stages between transcription and final assembly, interfering with the assembly of channel proteins, designing inhibitors that compete with the antibiotic for efflux, disrupting the energy source, and blocking the efflux channel (Pages and Amaral, 2009). Deletion of genes involved in expression of porins has been shown to increase antibiotic resistance (Rodrigues et al., 2011). Therefore, strategies combining overexpression of porins used by the antibiotic to penetrate the cell with downregulation of efflux pumps may allow the antibiotic to overcome existing resistance mechanisms.

## Conclusion

Functional metagenomic selections are a powerful technique for high-throughput characterization of the transferable resistome encoded by environmental and human-associated microbial communities, which have the potential to provide human pathogens with resistance genes through HGT. The advantages of this technique over traditional culture- and sequence-based screens are underlined when considering novel resistance mechanisms. Because resistance genes are selected on the basis of function regardless of sequence, the functional metagenomic selections reviewed above identified numerous genes with sequences highly divergent from other members of their class, as well as novel resistance mechanisms that would not have been recognized as resistance genes based on sequence alone. Conversely, they confirm antibiotic resistance function in putative drug transporters, which are frequently annotated as such regardless of true resistance ability, and permit the accurate annotation of resistance genes typically associated with other cellular functions. They have also led to the discovery of confirmed bifunctional enzymes, with broad resistance spectrums resulting from the fusion of two resistance genes. By providing a broader understanding of the resistance mechanisms currently in existence, functional metagenomic selections will aid the production of new antibiotics less susceptible to existing resistance mechanisms, as well as alternative strategies for combating antibiotic resistance.

### Conflict of interest statement

The authors declare that the research was conducted in the absence of any commercial or financial relationships that could be construed as a potential conflict of interest.
